# The Influence of Trust on Creativity: A Review

**DOI:** 10.3389/fpsyg.2021.706234

**Published:** 2021-08-13

**Authors:** Yan Chen, Cheng Yu, Yuan Yuan, Fang Lu, Wangbing Shen

**Affiliations:** ^1^School of Psychology, Nanjing Normal University, Nanjing, China; ^2^School of Education, Nantong University, Nantong, China; ^3^East China Campus, China Construction Bank University, Changzhou, China; ^4^School of Education, Nanjing Normal University of Special Education, Nanjing, China; ^5^Jiangsu Provincial Key Constructive Laboratory for Big Data of Psychology and Cognitive Science, Yancheng Teachers College, Yancheng, China; ^6^School of Public Administration and Business School, Hohai University, Nanjing, China

**Keywords:** trust, creativity, interpersonal trust, dispositional trust, team communication

## Abstract

Examining the trust-creativity relationship is important to promote creativity and organizational innovation. The goal of this study is to investigate how trust influences creativity by summarizing existing findings of diverse empirical studies. The impact of trust at different levels on creativity primarily manifests in three ways: (1) individuals' cognition- and affect-based trust has a positive effect on creativity together with the role of trust-derived perspective taking in creativity; (2) interpersonal trust helps enhance the joint creativity of an entire group via mediators such as team communication and commitment together with trust-evoked safety and the motivation to risk proposing, sharing, accepting or adopting uncommon ideas; (3) group trust has a positive, mostly indirect effect on creativity via mediating variables such as collaborative culture/climate and team communication. Potential implications and avenues for future research are also discussed.

## Introduction

Trust and creativity are the two fundamental elements that drive the reproduction and development of human society. The two features are important driving forces behind the sustainable development of enterprises, societies, and nations, and they are valuable assets of the human spiritual world. With rapid economic and technological development, creativity is the key factor involved in technological innovation and the scientific revolution and in improving the core competitiveness of enterprises (Zhou and Shalley, 2003; Anderson et al., 2004). Creativity typically manifests in human activities at different levels, ranging from everyday life to advanced technological industries. Trust is a key aspect of various social interactions and an important condition triggering efficient organizational collaboration and performance (McLean, 2005). Trust also plays an increasingly important role in the sharing economy, in online trade, and in business negotiations, particularly in situations involving risk, uncertainty, and interdependence (McKnight and Chervany, 2001), and it has been widely examined in many disciplines, including but not limited to positive psychology (Nakamura and Csikszentmihalyi, 2003; Charyton et al., 2009), anthropology, and sociology (Beldad et al., 2010).

In recent decades, psychologists have conducted numerous studies and in-depth discussions of these two issues and have accumulated a large number of meaningful results. With regard to trust, some researchers have argued that although trust is rich in connotations and important for any organization (Rousseau et al., 1998; Gong et al., 2017), it can be defined at the individual, interpersonal (or interindividual), and group levels and often includes cognition- and emotion-based trust (McAllister, 1995), competence, goodwill, and integrity (Mayer and Davis, 1999). In terms of creativity, no consensus has been reached on its definition. According to the “standard definition,” however, creativity, is conceptualized as the ability to produce something that is novel and useful (Sternberg and Lubart, 1996).

Although research on trust and creativity has been fruitful, an examination of their possible relationship, especially of the potential influence of trust on creativity, has been lacking. However, perhaps due to the initiatives of innovation-oriented national development strategies, more significance is being attributed to trust and creativity, resulting in increased research regarding the roles of trust in creativity in recent years and emerging critical findings in this field. In addition to a growing number of journal articles, several books (e.g., Xiaoge and Ming, 2021) and chapters (e.g., Jo and Lee, 2012; Wu et al., 2016) have been devoted to this topic. While these studies have made important theoretical and conceptual advancements, particularly in understanding trust and creativity as well as their potential association, the associated literature, which is mostly fragmented and scattered across various disciplines, does not provide an integrated and coherent understanding (McEvily et al., 2003). This may be partially the case because most research considers trust to be a variable or construct of existing theories of organizations, resulting in inconsistent and diverse findings on the hierarchy of trust. Anecdotally, researchers tend to use different approaches to trust to satisfy the idiosyncratic requirements and purposes of a particular study. However, we simply do not have an accurate and clear sense of the extent to which consistency across various studies exists in the impact of trust on creativity, since no systemic literature review on this issue is available.

Without an evidence-based review of the influence of trust on creativity, advancing our understanding of effects, roles, and potential mechanisms of trust in relation to creativity would be impaired and fragmented. There is also no way of evaluating how diverse or integrated the literature is, the mechanism by which trust influences creativity, and the specific role of different types of trust in creativity. To our knowledge, no systematic review has evaluated the effect of trust on creativity. Accordingly, to establish a comprehensive understanding of the essence of creativity and the potential role of trust in creativity, this work attempts to integrate available studies from an integrated research perspective.

This review makes at least three contributions. First, we offer a careful assessment of the current state of research approaches used in determining the role of trust in creativity, especially organizational creativity. Our evaluation of the degree of fragmentation and convergence across studies is conducted by examining the effect of trust on creativity. Second, as mentioned above, trust is a multidimensional construct that can be found in a single person (individual dispositional trust), between two individuals (interpersonal trust), and within groups of individuals (group trust; Brattström et al., 2012). Accordingly, we profile three typical and primary approaches and summarize the separate influences of individual, interpersonal and group trust on creativity. Third, the present review could be used to construct guidelines for investigating the impact of trust on creativity and for nurturing organizational creativity in the future. Taken together, the presented results will be of important value to future research on trust and in cumulating useful knowledge on trust and creativity development. Our review focuses on studies that examine the influence of trust on creativity across the individual and/or group levels, including studies on dispositional and situational trust (see McEvily and Tortoriello, 2011). The remainder of this paper is organized as follows. We first provide a definition of trust and discuss the role of individual trust in creativity. Then, we evaluate the role of interpersonal trust in creativity. Next, we illustrate the impact of group trust on creativity. Finally, we summarize the impact of different types of trust on creativity with proposed directions for future research.

## Methodology

The current study is framed as a narrative literature review. Different from a meta-analytical investigation, a narrative literature review offers many advantages in the analytical integration of different studies adopting various perspectives or distinct methodologies (Samuelsen et al., 2012) without requiring all selected studies to align with the same rigorous design of a systematic empirical evaluation (Green et al., 2006). Following prior studies (e.g., Mahmood et al., 2014), a standardized methodology focused on literature searching and inclusion was employed to execute an integrated analysis of the role of trust in creativity. Specifically, to identify publications specific to the role of trust in the field of creativity and collect publication records on the creativity-trust relationship, multiple combinations of relevant search terms (e.g., creativity, convergent thinking, creative, divergent thinking, innovation, and trust) were devised to search through several comprehensive databases. We searched for literature between April and May 2021 without specifying periods of publication. An established stepwise approach was followed to search for and screen out potentially relevant studies in this integrated analysis.

To identify as many relevant papers as possible, a comprehensive literature search was first conducted by searching for relevant papers (1) published in all international creativity journals; (2) listed in multiple online electronic databases, including the Springer, Google Scholar, Web of Science, CNKI, EBSCO, Elsevier, and ProQuest Digital Dissertations databases; (3) published in a trust-specific journal, e.g., *Journal of Trust Research*; and (4) accessible from other information sources, including the citation records and references of the preselected papers. To unbiasedly identify the literature on the role of trust in creativity, we also analyzed master's theses and doctoral dissertations that satisfy the literature inclusion criteria. We focused on each paper's subject, title, abstract, and keywords without searching for the target words in the full text (due to the large number of studies pertaining to trust and our limited resources).

**Figure 1:**
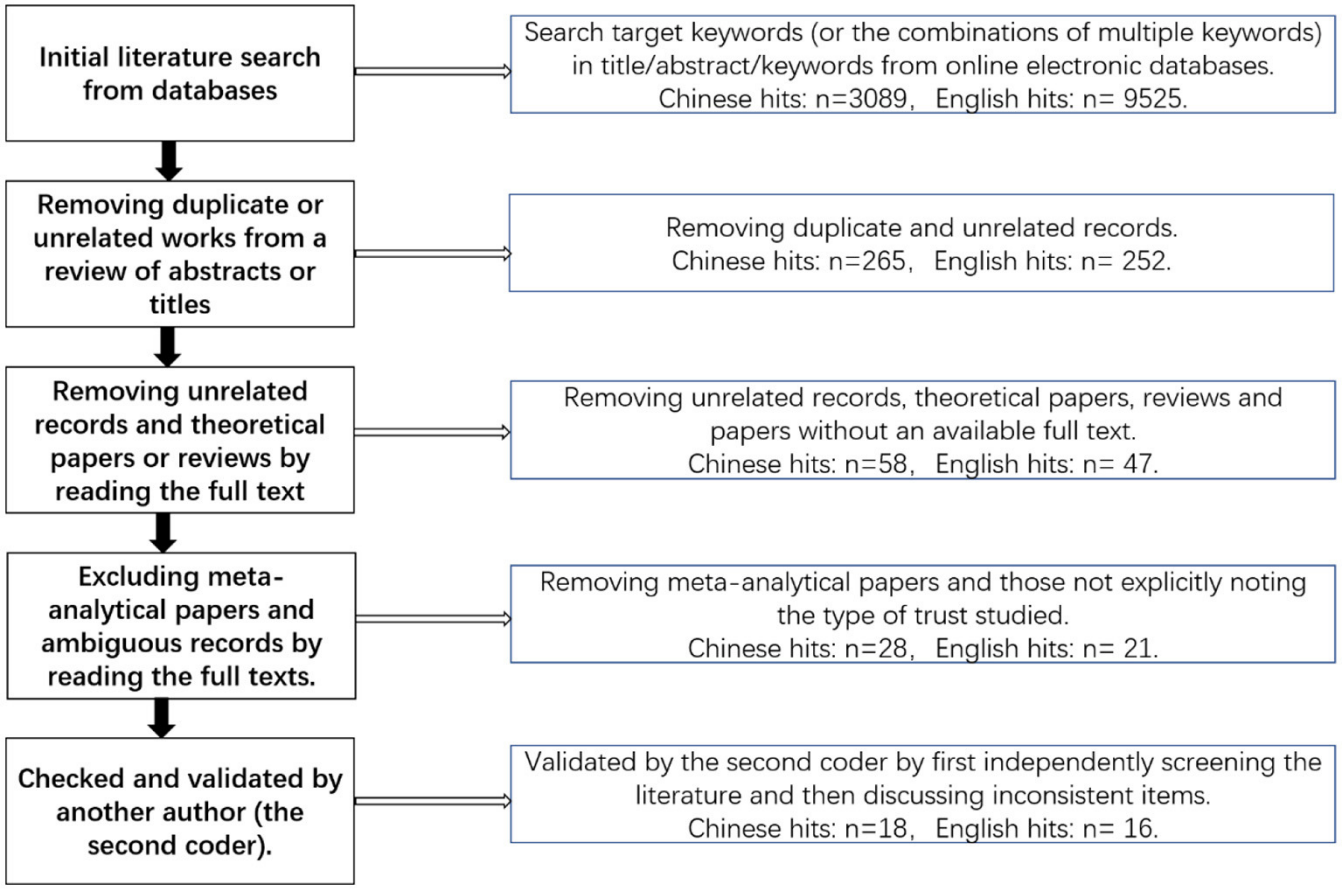
A schematic illustration on the process of literature inclusion and exclusion.

In addition, we developed literature inclusion and exclusion criteria. All reviewed studies are written in Chinese or English, providing empirical (correlational or experimental) data results on the relationship between trust and creativity. Papers were excluded according to the following criteria (see Figure 1): (1) those not addressing the role of individual/interpersonal/group trust in creativity or not explicitly stating which form of trust was measured; (2) those not theoretical, meta-analytical, or conceptual in nature (e.g., perspectives, theoretical analyses, commentaries, dialogs, qualitative evaluations, editorials, book reviews, literature reviews, and newspaper/magazine articles); and (3) those for which full texts were not available. Supplementary Table 1 lists the hit records and shows the impacts of individual, interpersonal, and group trust on creativity.

## The Impact of Individual Trust on Creativity

Developed by psychologists in the 1950s, the concept of trust has since be explored in many other areas (Smith, 2010), such as sociology, management, and marketing. Trust can be conceptualized as a willingness to be vulnerable to others (Mayer et al., 1995) and has long been treated as a stable dispositional or individual difference variable. (Gefen, 2000, p. 728) argued that “disposition to trust is a general, … not situation specific, inclination to display faith in humanity and to adopt a trusting stance toward others.” Following the integrative model proposed by Schoorman et al. (2007), trust is a function of one's disposition or propensity to trust people in general together with one's perceptions of trustworthiness regarding specific other(s) in terms of moral integrity, personal benevolence, and ability. Thus, some persons are “more trusting” of others, and a person with high levels of dispositional trust tends to be more likely to trust others than a person with low levels of dispositional trust. Likewise, initial levels of trust found in an interaction do not have at a starting point of zero (i.e., no trust) but vary from person to person (Kramer, 1999). Several studies have empirically revealed and documented the effects of dispositional trust. As a dispositional trait, individual trust takes two primary forms: cognition- and affect-based trust (McAllister, 1995). Cognition-based trust, which serves as an “uncertainty reducer” in facilitating interpersonal communications, refers to the extent to which partners' or others' competence and responsibility is positively expected (McAllister, 1995). In contrast, affect-based trust, which largely builds on emotional investment, genuine care, concern for partners' welfare and relevant reciprocation belief, refers to the extent to which an individual expects her/his partners to express care and concern toward her/him (McAllister, 1995).

Trust involves having confidence in others and their good intentions even when risks are involved and therefore is a fundamental facet of social interactions. First, people with higher trust scores are unlikely to deceive others in interpersonal relationships and are highly likely to give others the benefit of the doubt unless there is clear evidence that the other party cannot be trusted (see Lewicki and Robinson, 1998). In addition, those who are trusting appear to be more trusted and popular among others (Rotter, 1967). Some scholars (e.g., Driscoll, 1978) argue that the effect of trust as a personality trait can be “washed out” given a person's level of trust within a defined context; certain trust states may mediate the relationship between generalized trust and trusting behavior. According to Panteli and Sockalingam (2005), trust implies that others trust the language, actions, and decision-making abilities of others they can trust, and as a result, they also try to take action. In this regard, trust enables cooperative behavior, facilitates collaboration-based organization forms, reduces interpersonal conflict, encourages the rapid formulation of *ad-hoc* work groups, and improves the creativity of individuals or teams (e.g., Chen et al., 2008; De Clercq et al., 2009; Tsai et al., 2012). The impact of trust on creativity primarily manifests in the roles of cognition- and affect-based trust in creativity.

On the one hand, cognition-based trust enables helping behavior (e.g., Colquitt et al., 2012), reduces personal uncertainty for creative individuals, and allows these individuals (especially those who are open to experience) to believe that their peers can help them effectively complete their work. Creativity, which is highly associated with risk taking and sometimes termed a risky investment process, is often conceptualized as a process of finding solutions to problems, deficiencies, knowledge discrepancies, missing components, and dissonances (see Sternberg and Lubart, 1996; Tyagi et al., 2017). Given that creativity involves high levels of uncertainty and risk (Shen et al., 2018) and as radical creativity in particular entails a substantial departure from existing work procedures and techniques, this form of individual trust may be helpful in increasing creativity. Additionally, cognition-based trust is useful in facilitating communication between a person and her/his trusted peers (McAllister, 1995), obtaining useful feedback and even challenging critique (beneficial to radical innovation) from peers (Zhou, 2003), and evoking a belief that peers can provide social support and help in solving problems as necessary and in problem solving (Madjar et al., 2002, 2011). All of these resources help facilitate the development of a creative solution that is safe and reliable (Madjar et al., 2002, 2011). Poor cognition-based trust in others, including colleagues, would make it impossible for problem solvers or innovators to put themselves in situations where they can obtain valuable resources for creative thinking. From the interactionist perspective, Xu et al. (2016) found that cognition-based trust in one's colleagues is positively related to perceived creativity, implying that cognition-based trust generally helps employees generate creative ideas. In contrast, affect-based trust can build mature interpersonal relationships and encourage safe emotional attachments that may relieve employees' anxieties toward taking greater risks associated with creativity. Affect-based trust does not seem to be directly related to creativity, but employees may feel more confident about taking risks when they have affect-based trust in their colleagues. In situations where strong affect-based trust is present, individuals feel particularly able to seek and respond to feedback from their peers. Even if constructive feedback contains some criticism, it may be welcomed in light of positive emotional connections maintained with peers or colleagues. Therefore, affect-based trust can help individuals experience a sense of security and encourage them to pursue creative solutions, especially those that are radically creative (Madjar et al., 2002). Indeed, some studies have reported that trust induces creativity and/or innovation when communication is open and the environment is supportive, tolerant, and friendly (Simons and Peterson, 2000; Martins and Terblanche, 2003; Dakhli and De Clercq, 2004). In fact, generating a new idea requires practicing divergent reasoning, identifying many possible solutions, communicating with others, modifying alternatives, and choosing clever solutions to new problems (Zhou, 2003). Trust is an important trait that increases individual creativity, idea generation, information sharing, and openness (Dirks and Ferrin, 2002; Den Hartog, 2003; Bidault and Castello, 2009).

Trust tends to be interpreted as involving uncertainty (e.g., Mayer and Mussweiler, 2011), which is often considered the default state or trigger present when one feels that specific others are “safe” in the sense of being dependable, having no malevolent motivation, and being able to assist. In this regard, a state of trust (distrust) may resemble more general positive (negative) mood states. According to Schwarz (2012), people often draw on their moods as a cue of the nature of their situation: Good moods signal a benign situation, whereas bad moods signal a problematic situation. Moreover, because thinking processes are tailored to the given situation, experiencing good vs. bad mood states can lead to different processing strategies (Schwarz, 2012). Cognitive tuning theory (Friedman and Förster, 2000) and accumulated empirical evidence reliably show that a positive mood can motivate individuals to adopt a risky, explorative, and heuristic thinking style and then lead to the development of creative or insightful solutions. In contrast, negative moods can trigger a more conservative, careful, deliberative, and analytical style and are more likely to result in the generation of routine or analytical solutions. Similar to mood states, trust/distrust can shape the types of processing strategies people utilize based on the challenges and opportunities of the situations that trigger them. Because creativity is a non-routine behavior that involves “thinking outside of the box” (Nusbaum and Silvia, 2011) and often requires adopting an unusual perspective to challenge obvious responses or mental sets, trusting individuals may be better able to master multiple frames by thinking from another person's point of view. Accordingly, creativity is a risk-taking behavior that is usually seen as deviant behavior in standard work settings. A perceived threat from a leader can be induced by the proposal of new ideas and the potential fear resulting from a high probability of idea failure. Employees who trust their leaders tend to exhibit creative behavior through the adoption of a heuristic approach.

Perspective taking is an important ability involved in problem-solving activities and can further enhance individual creativity, especially divergent thinking and cognitive flexibility. An object generally has more than one property and multiple functions that can be seen quite differently according to one's perspective (Naghavi and Nyberg, 2005). An object considered or viewed from one specific perspective is seen as having one particular superordinate even though it could have many. Different perspectives on the same object cause different properties to be highlighted, which further triggers different uses of the same object. In other words, viewing an object from a new perspective can help in perceiving a novel function or meaning of the object (Shen et al., 2017). The process of creativity, especially divergent thinking and solving insight tasks, is needed because the problem solver must move away from common or high-frequency responses and highly activating mental sets to view a problem from a unique or unusual perspective or identify a constrained new search space in which the solution resides (Chermahini and Hommel, 2012; Shen et al., 2018). In this regard, perspective taking benefits creativity by allowing for many distinct or novel perspectives. Additionally, the impact of trust on creativity may not be direct; instead, the effect may be indirect and mediated by moderators such as openness to experience. Several studies (e.g., Madjar, 2008) have reported that openness to experience may strengthen the role of individual trust in creativity because persons who score high in this trait tend to demonstrate open-minded thinking and to not pay attention to markers of uncertainty; therefore, when such individuals trust their leaders, they exhibit high levels of openness to experience and more creativity.

Taken together, most available studies tend to support the influence of individual trust on creativity. This influence typically manifests in the facilitating roles of cognition- and affect-based trust in creativity across the individual and group levels together with the positive association between perspective taking derived from individual trust and creativity, particularly for divergent thinking and insight. Additionally, the positive effect of individual trust on creativity can be strengthened or weakened by moderators such as openness to experience, organizational commitment, creativity measurement, and leadership style.

## The Impact of Interpersonal Trust on Creativity

Trust that originates within the individual is not only a cornerstone of a collaborative climate but is also inherently relational and interpersonal in essence (Zaheer et al., 1998). In contrast to individual trust, a disposition involving assuming the general trustworthiness of others, the form of relational trust present is related to the counterpart in the dyad and is based on experiences and interactions with a particular exchange partner (Ring and Van de Ven, 1992). That is, trust can be divided into (1) individual trust primarily influenced by an individual's growth trajectories, personality type and cultural background and (2) interpersonal trust born from human interaction and heavily reliant on whether an individual's characteristics are trustworthy (Cerne et al., 2014). Trust among people is essentially interpersonal; it involves an individual being willing to be defenseless to the other party and having positive assumptions regarding the other person's intentions, actions, or behaviors (e.g., Mayer et al., 1995; Rousseau et al., 1998). Such trust may form from the propensities of the trustor, but it is not purely a personality trait but a condition that is also influenced by the given context, interactions with the other person, and individual differences. Therefore, interpersonal trust is primarily psychological (Rousseau et al., 1998), but it is actually interactive in that both parties trust and are trusted (individually in the positions of trustors and trustees) to engage in mutually beneficial exchanges.

Trust is a facet of all relationships and interactions and is recognized as a crucial ingredient in building trustworthy organizational behavior and workplace collaboration (Dirks and Ferrin, 2001), resulting in a variety of positive effects. All 43 empirical studies reviewed by Dirks and Ferrin (2001) show that interpersonal trust benefits individuals or their organizations. These expected benefits include improved communication, more organizational civic behaviors, reduced competitive behavior in negotiations, heightened group performance, alleviated conflict, and enhanced job satisfaction. Creativity has been emphasized and discussed in various fields. For individuals and organizations to remain sustainably competitive, constant innovation is required, and employee creativity initiates such innovation. To unleash organizational creativity and increase organizational innovation, individuals, and organizations need to induce creativity through personal effort or systematic organizational mechanisms. Employees are trusted, and experiences of trust support their activities. Employees are aware of the need to be trusted and to trust in their organizations (e.g., leaders and managers) to minimize risk (Guchait et al., 2012), and trust is an important dimension of organizational creativity. Rather, scholars are greatly interested in the supportive role of interpersonal trust in promoting employee creativity. Theoretically, Prati et al. (2003) related interpersonal trust to creative performance. Empirically, Weng (2014) found that trust developed based on the relationships between supervisors and subordinates is the primary motivator of an innovation climate. Klimoski and Karol (1976) reported that trusted teams outperform less trusted teams in terms of creative problem solving. In a study of 82 student teams, Barczak et al. (2010) also documented a positive association between team trust and creative performance. In other words, interpersonal trust can improve organizational creativity and/or innovation. Building on existing studies, Bidault and Castello (2009) constructed a dual-pathway framework to explain the effect of interpersonal trust on creativity. First, mutual trust between partners of collaborative projects influences partnership creativity; therefore, interpersonal trust can promote collaborative creativity. Research indicates that the more advanced a team's level of experience is, the more innovative its output will be (Taylor and Greve, 2006). Trust in teammates is considered to be the foundation that allows team members to freely share knowledge, explore, and make the utmost contributions to the success of their tasks. Trust seems to create a more open, supportive, and tolerant environment and reduce hostility and competitiveness. This is particularly true when one must collaborate or wants to achieve creative outcomes (Barczak et al., 2010). In addition, trust affords team members more freedom, which can trigger the development of new ideas and mitigate conflicts. All of these factors should result in a higher level of creativity. It has also been shown that trust in partners is associated with partnership commitment. Prior studies have shown that while low levels of trust can be abused by a partner through opportunism and “free-riding” behavior, leading to low levels of commitment, high levels of trust increase commitment and have a positive impact on creativity or innovativeness. When the two effects are combined, innovation leads to creativity, as creative ideas are implemented through the use of resources committed by both partners, leading to innovation.

Moreover, interpersonal trust is a fundamental factor involved in dynamic collaboration and continuous knowledge sharing, which may have moderating and indirect effects on creativity (Huang et al., 2006). On the one hand, if team members trust each other, they cannot be defenseless and can dedicate more energy to creating and discovering rather than defending themselves. In this situation, trust is key to uniting employees, which facilitates the generation of creative solutions or creative collaboration. More generally, negative expectations of competence and reliability (i.e., low cognitive trust) make individuals less attractive as exchange partners or “sounding boards” for new ideas and reduce team members' willingness listen to these individuals' alternative ideas and perspectives. In turn, these effects dampen creative collaboration. In support of this reasoning, Barczak et al. (2010) empirically revealed that interpersonal trust can positively influence organizational creativity via a collaborative culture or cognitive trust. On the other hand, recent research linking social networks to creativity emphasizes that creativity involves a social process (Perry-Smith, 2006) and that fluency and openness in sharing diverse and novel ideas are key to creative performance. Trust generated from the emotional bonds between individuals (i.e., affect-based trust) enables creative collaboration by motivating people to carefully listen to, share and understand others' alternative perspectives and by resolving potential misunderstandings arising from cognitive gaps in problem representation that tend to occur in interactions. That is, affect-based trust is a means to facilitate communication and generate creative or new ideas. By understanding and appreciating different perspectives, when individuals understand and appreciate perspectives that differ from their own, individuals with high levels of affect-based trust can successfully manage relevant frictions, engage in constructive debate, and use essential differences to generate creative solutions. In this regard, affect-based trust creates a conduit allowing for the two-way communication of new ideas and motivates individuals to better understand diverse perspectives, facilitating creative collaboration, especially between culturally different individuals. Chua et al. (2010) reported that affect-based trust is particularly relevant for sharing new ideas in a manager's job network. In a large-scale investigation by Monge et al. (1992), this link between group communication and team innovation was replicated. From a review of individual-, team-, and organizational-level factors related to creativity, Shalley and Gilson (2004) found that leaders should promote communication between team members if they wish to foster creativity or strengthen the positive relationship between team communication and creativity.

Furthermore, trust in interpersonal relationships reflects a willingness to accept vulnerability based on positive expectations of another person's intentions and actions. Given that trust plays an important role as a “social lubricant” and has been established to alleviate conflicts within an organization, individuals can interact with each other in any exchange condition and in dialog with a partner who exhibits confidence and distrust. Therefore, the effect of interpersonal trust is considered to be particularly effective in situations where the intentions of others are somewhat ambiguous. However, among other organizational, interpersonal, and personal factors that may alleviate interpersonal conflict, trust is associated with exchanges between individuals occurring at the same hierarchical level. In short, trust in others can play a positive role in an individual's willingness to act innovatively by creating a sense of connection (Carmeli and Spreitzer, 2009). Overall, affect-based or emotional trust is considered to be particularly important in interpersonal relationships. Affect-based trust buffers interpersonal anxiety, which often impedes close cooperation (Stephan et al., 1999; Thomas et al., 2009), especially the sharing of new ideas across different cultures. Notably, affect-based trust emphasizes the kind of resilient, flexible, and adaptive relationship that allows managers to respond to market changes in a timely manner. Cognitive trust may occur when the internal and external environments are relatively stable, while emotional trust may be advantageous when the environment is more volatile and it is difficult for managers to make specific plans and forecasts for business operations, requiring them to be flexible in their strategic direction (Zhang, 2014).

In summary, the role of interpersonal trust in facilitating creativity, which brings something both new and useful to an organization, is primarily such that, consistent with the dual-pathway framework proposed by Bidault and Castello (2009), interpersonal trust not only facilitates joint creativity but also enhances the creativity of an entire group via mediators such as a collaboration-based shared mind, systematic efforts, and team communication or commitment. In addition, individual willingness or motivation induced by interpersonal trust may be critical in facilitating organizational creativity through the sharing of diverse, distinct, and sometimes unique ideas or perspectives.

## The Impact of Group Trust on Creativity

Trust in a team context, namely, team trust, refers to the belief that one's teammates have good intentions and confidence in the capabilities and character of their partners or colleagues, which is a key basis for constructing a friendly and harmonious environment and climate for teamwork and achieving better outcomes. With the expansion of team-based work, the role of trust in work teams has gained momentum. Research has increasingly accumulated, particularly at two specific levels of analysis—interpersonal trust between individual members at the individual level and group trust (sometimes termed team trust) shared among members at the team level. In this regard, group trust can be seen as a special form of interpersonal trust that involves multiple agents beyond the two agents of mutual trust and the members of a group. In support of this idea, McAllister (1995) adopted the term interpersonal trust to conceptualize trust among team members. Compared to individual trust, trust in a group setting, typically referred to as group (team) trust, often involves one or multiple targets for a given trusting individual. According to Cummings and Bromiley (1996), group trust in a team means that “individuals and groups strive in good-faith efforts to act in accordance with all promises regardless of (a) explicit or implicit, (b) sincere in any negotiation prior to such a promise, and (c) does not overuse others even if there is an opportunity” (p. 303). This definition of trust in a group is closely related to the definition of trust in an individual and refers to general feelings of trust in other members of a given group.

Creativity is often defined as the origination of ideas, problem solutions, and visions that are both novel and appropriate and usually appears as an interaction between a person and the given situation (Scott and Bruce, 1994; Amabile, 1997). In other words, creativity depends on organizational conditions such as the freedom of idea generation, team characteristics, and support and encouragement from superiors (Amabile, 1998). Trust is widely supported as an important precursor to performance, and few studies have examined the role of group (team) trust in individual and/or team creativity. According to Whitener et al. (1998), teams need more trust (than individuals) because they require a high degree of interdependence to complete their tasks. In general, a team in which members have various goals, perspectives, and backgrounds is more likely to result in misunderstanding, conflict and miscommunication among teammates even though such a team is considered more creative. A high level of team trust allows members to be better able to focus, communicate and support each other and increases team creativity. When team members trust each other, they are more likely to work closely together, share knowledge and allocate resources to common goals (Wicks et al., 1999; Dirks and Ferrin, 2001). Consequently, teammates become more motivated and pursue their common goals to achieve more creative outcomes (Jaskyte, 2008). Research shows that team members tend to be very creative and innovative when interpersonal communication, support, and purpose clarity are at high levels (Jaskyte, 2008). Barczak et al. (2010) surveyed a team of 82 students and found a positive relationship between team trust and creative task performance. Both team trust and a collaborative culture can support more effective communication, information sharing, focus, and cooperation, which leads to more creative endeavors. Research on a sample of 273 master's students from the University of Science and Technology of China shows that team trust has a reliable impact on team creativity by promoting team communication.

High levels of trust allow teams to work smoothly and reach their goals (Wicks et al., 1999). Trust helps forge interdependent relationships and facilitate creative collaboration. Without trust, political behavior emerges within a team, pitting individuals with different perspectives against each other, which would further interfere with or undermine the efforts of other team members. In other words, trust is critical to constructive and positive collaborative relations (Adler, 2001). However, group trust is dynamic in that early trust may be different from later or mature stages of trust; early trust may be based on abilities and tends to be more cognitively based while only later drawing on benevolence (Knoll and Gill, 2011) and becoming more affect based, which might be established by fostering greater communication among team members. In general, initial trust among team members is thought to be based on two factors: (1) demographic similarity judgments (e.g., Costa, 2003) and (2) information about team members. This information is conveyed through the first communication among teammates. Through this relatively short communication process, team members communicate information to determine the three factors involved in building trust: teammate competence, goodwill, and integrity (character) (Mayer et al., 1995). As group trust evolves, a collaborative culture, an important prerequisite to team creativity, is gradually formed, which can affect how team members interact and collaborate. Barczak et al. (2010) present a more nuanced and complex scheme of the antecedents of “team creativity.” The authors find that team trust helps build a collaborative culture that leads to higher levels of team creativity. From a survey of 82 student teams at a large university, Barczak et al. (2010) found that team trust fosters a collaborative culture that enhances team creativity. A trusting environment involves connecting with colleagues, team spirit and cooperation, the ability to take risks, greater tolerance, and receptivity to diverse ideas and perspectives. Thus, teams that trust each other are more likely to work closely together and engage in meaningful reciprocal behaviors in addressing problems and issues, which is essential to creating effective outcomes.

Similarly, cognitive trust can increase team creativity through the moderating roles of collaborative culture or team communication. Creativity allows teammates to solve problems and leverage opportunities by integrating various perspectives or divergent thoughts developed by their partners or team members (Tu and Lu, 2013), wherein team trust enables individuals within a group or team to feel free to share information, explore, contribute their ideas and exhibit an increased willingness to assume the risks of creativity without a fear of failure (Barczak et al., 2010; Shen et al., 2018). That is, teams with high levels of cognitive trust will easily be perceived to include members with strong functional and interpersonal capabilities that can create a secure attachment or feelings of safety from which the team can collaborate, jointly make decisions, take risks and share ideas without fears of criticism, invoking more creative solutions to their tasks. Consistent with Bierly et al. (2009) and Barczak et al. (2010) also reported that team trust builds on individual confidence and belief that the actions of members are beneficial to joint and/or team creativity.

To conclude, the number of available studies examining the effect of group trust on creativity is still very limited, with most showing an indirect effect of trust on creativity. Building on previous studies, group trust positively influences organizational creativity, especially team creativity, through a collaborative culture (climate) and through harmonious and positive team communication cultivated by cognition- and affect-based trust, which in turn enables teammates to feel free to share information, explore, contribute their ideas, and exhibit an increased willingness to assume the risks of investing and engaging in creativity.

## Concluding Remarks

Trust can facilitate creativity. In this review, from the perspective of the trust hierarchy, the positive effect of trust on creativity or innovation was reviewed and summarized. Studies of trust typically focus on the roles of individual, interpersonal, and group trust in individual and/or team creativity in organizational settings. Our research makes an important contribution to the literature by offering the first review carefully summarizing the role of trust across different levels of individual and/or team creativity in organizational settings and providing key insights to future studies in this field by demonstrating the positive role of trust in organizational creativity and innovation and the potential mechanisms by which trust influences creativity. Several studies have suggested that trust can be divided into different categories, resulting in the differentiation of goodwill and general trust, dispositional and state trust (e.g., Ross and LaCroix, 1996) and cognition- and affect-based trust (Schaubroeck et al., 2011). Future studies should make greater efforts to systemically investigate and isolate the roles of such different types of trust in individual and team creativity. Nevertheless, it should be noted that several studies have reported that high levels of team trust might have dysfunctional consequences for team creativity. This effect can be accounted for or moderated by the group-centrism perspective, which seems worthy of further investigation. In addition to consolidating the positive impact of trust on creativity, the specific mechanism by which trust facilitates creativity should also be examined, as this would further deepen understanding of the relationships that form between trust and creativity and organizational innovation through nurtured organizational creativity and trust. Additionally, future studies could adopt well-developed technologies, such as behavioral experiments, or interbrain measures, and meta-analytical approaches with large sample sizes to investigate and empirically determine the roles of individual, interpersonal, and group trust and different orientations of trust such as trust toward leaders or trust toward subordinates in creativity within or beyond organizational settings.

## Author Contributions

All authors listed have made a substantial, direct and intellectual contribution to the work, and approved it for publication.

## Conflict of Interest

The authors declare that the research was conducted in the absence of any commercial or financial relationships that could be construed as a potential conflict of interest.

## Publisher's Note

All claims expressed in this article are solely those of the authors and do not necessarily represent those of their affiliated organizations, or those of the publisher, the editors and the reviewers. Any product that may be evaluated in this article, or claim that may be made by its manufacturer, is not guaranteed or endorsed by the publisher.
