# Supplementary material for: The Influence of Trust on Creativity: A Review
Source: Front Psychol. 2021 Aug 13;12:706234. doi: 10.3389/fpsyg.2021.706234 (PMC8415111; doi:10.3389/fpsyg.2021.706234)
Supplement: Supplementary file 1 [file Table_1.docx]

**Table 1** Summary of the literature on individual, interpersonal, and group trust

| **Number** | **Source** | **Variables** | **Creativity & trust measures** | **Type** | **Sample** | **Main findings** |
| --- | --- | --- | --- | --- | --- | --- |
| 1 | Wang, 2016 | Personal trust, knowledge sharing, and innovative behavior. | The individual trust measure was modified from Kuhnert's (1992) organizational trust scale, which includes three dimensions: trust in the system, trust in the leader, and trust in colleagues. The innovative behavior measure was modified from Scott & Bruce (1994) and Kleysen & Street (2001) and includes two dimensions: idea generation and idea execution. | Individual | 203 persons | Personal trust is significantly and positively related to innovative behavior (system trust and trust in leadership are both not significantly related to the production concept dimension, while all other dimensions are significantly related to each other), and knowledge sharing plays a fully mediating role in the positive influence of personal trust on innovative behavior. |
| 2 | Liu, 2017 | Individual trust, work engagement (energy, dedication, and focus), and innovative employee behavior. | The individual trust measure was modified from Kuhnert (1992) and includes three dimensions of trust in the organization, in the leader, and in colleagues; the employee innovation behavior measure was modified from Scott & Bruce (1994) and Kleysen & Street (2001). | Individual | 340 participants | Individual trust is significantly and positively related to innovative behavior, and work engagement partially mediates the relationship between group trust and employee innovative behavior. |
| 3 | Ge, Wu, & Zhao, 2016 | Learning orientation, team trust (including cognitive and emotional trust), and corporate innovation performance. | A trust scale was adopted from McAllister (1995) and a measure for innovation performance was adopted from three published studies. | Individual | 2018 members from 210 technology-based companies in Jiangsu | Learning orientation positively affects innovation performance; cognitive team trust can play a partially mediating role between learning orientation and firm innovation performance, and emotional team appointment has no direct effect on firm innovation performance. |
| 4 | Javed, Rawwas, Khandai, Shahid, & Tayyeb, 2018 | Openness to experience, trust in leaders, ethical leadership, and employee creativity. | A nine-item employee creativity measure developed by Tierney Farmer and Graen (1999) was applied.  Trust: Employees rated five “trust in leader” items from Anand, Chhajed, and Delfin (2012). | Individual | 205 supervisor–subordinate small textile firms across Pakistan | Trust in leaders has a positive relationship with creativity and mediates the effects of ethical leadership on creativity. In addition, trust in leaders and openness to experience have a shared effect on creativity. |
| 5 | Huang, Gino, & Galinsky, 2015, study 4 | Trust, sarcasm, conflict, and creativity. | In terms of trust, participants remembered the person they trusted the most, wrote this individual’s initials, briefly described the person’s face, and explained why they trusted this person. Under distrustful conditions, participants went through the same process but recalled the most distrustful (or least trusted) person they knew.  The Olive in a Glass problem was used to measure creativity. | Individual | 258 Americans | Study 4 found that when participants expressed sarcasm toward or received sarcasm from a trusted other, creativity increased but conflict did not. |
| 6 | Jo & Lee, 2012 | Trust, task complexity, intrinsic motivation, and employee creativity. |  | Individual | 365 persons | Individual trust is positively correlated with creativity, and the variable of intrinsic motivation is a moderator of the positive influence of trust on employee creativity. |
| 7 | Chua, Morris, & Mor, 2012, study 2 | Cultural metacognition, inter- vs. intracultural relationships, creative collaboration – sharing of new ideas, and trust. | Creative collaboration was measured with the following single-item measure: “How likely are you to share new insights or information with this person?”; trust was measured by the adapted measures of affect- and cognition-based trust from McAllister (1995). | Individual | 60 managers | Both cognition- and affect-based trust are positively correlated with creative collaboration with affect-based trust acting as a mediator of the positive impact of cultural metacognition on managers’ creative collaboration. |
| 8 | Jiang & Chen, 2017 | Trust, organization learning, and creativity. | A new model was developed and validated by the authors. | Individual | 373 supervisors or employees | The results show a positive effect of organizational trust on creativity. |
| 9 | Mayer & Mussweiler, 2011 | Trust (distrust), flexibility, and originality (creative idea generation). | (Dis)trust was primed situationally, whereas creativity was measured using an idea generation task. | Individual | 56 students | Trust has a negative relation to creativity measured as originality, and this association is partially mediated by flexibility. |
| 10 | Men, 2010 | Interpersonal trust, conflict (relationship, task), and creative performance; control variables: education level, gender, age, and role playing. | Interpersonal trust was measured through a scale adopted from McAllister (1995); creative performance was evaluated with a scale adopted from Ancona & Caldwell (1992). | Interpersonal | 199 participants | Both cognitive and affective trust are significantly and positively related to innovation performance; they both have significant direct effects on innovation performance and significant indirect effects through the partial mediation of conflict. |
| 11 | Wang, 2007 | Knowledge sharing, team cohesion, risk taking, and key team innovation behaviors: embedded team cooperation and communication behaviors, team innovation behaviors, and team decision-making behaviors. | The Trust in Innovation Teams in Higher Education Scale was adapted from McAllister et al. (1995) and measures task- and emotion-oriented trust. The Team Critical Innovation Behavior Questionnaire: Self-administered measuring team cooperation, communication, innovation, and decision-making was applied. | Interpersonal | 304 members of 39 innovation teams | Trust (task- and emotion-oriented) in university innovation teams significantly enhances team innovation behaviors. The integration of key behaviors (including innovation behavior dimensions) in university innovation teams plays a fully mediating role in the mechanism of action between trust and performance. |
| 12 | Bai, Wang, & Xi, 2008 | Transformational leadership, perceived organizational support, leader-subordinate exchange, organizational trust, individual trust, work performance, and innovative behavior. | Organizational trust was measured by a self-administered 4-item questionnaire; the competence dimension was adopted from the rational trust concept developed by McAllister (1995). Individual trust was adopted based on the definition of affective trust provided by McAllister (1995). The innovative behavior measure was adopted from Farmer et al. (2003). | Interpersonal | 179 supervisors and 1074 subordinates | Organizational and individual trust are both significantly and positively related to innovative behavior, but organizational trust can achieve a chain mediating effect on transformational leadership behavior. |
| 13 | Dong, Gao, & Ding, 2016 | Perceived fairness, interpersonal trust, and team knowledge-sharing behavior. | Interpersonal trust was measured on a self-rated scale adapted from published studies. Innovation team knowledge sharing behavior was measured as the indicated sharing behavior among members of innovation teams of three or more persons and its levels in universities and innovative enterprises; six questions were used to assess explicit and implicit sharing behavior. | Interpersonal | 230 persons from 30 teams | Teammates’ trust in an innovative group is positively correlated with member knowledge-sharing behavior, and all dimensions of member trust have a positive influence on teammates’ knowledge sharing behaviors. |
| 14 | Liu, 2013 | Interpersonal trust, knowledge sharing, knowledge integration, and innovation performance. | An interpersonal trust measure from McAllister (1995) measuring the three dimensions of cognitive, affective, and behavioral trust and an innovation performance measure from Scott et al. (1995) measuring the three dimensions of innovation ability, innovation behavior, and innovation outcome were adopted. | Interpersonal | 236 participants from 45 teams | Interpersonal trust in university research teams has a significantly positive effect on team innovation performance. Knowledge sharing and knowledge integration play a fully mediating role between team interpersonal trust and innovation performance. |
| 15 | Li, 2016 | Social relationships, team trust, and innovativeness. | Scales individually measuring trust and creativity were adopted from previously published studies. | Interpersonal | 253 persons | Competence trust and affective trust are significantly and positively associated with innovativeness, but only affective trust can mediate the role of social relationships in innovativeness. |
| 16 | Song, 2015 | Interpersonal trust, knowledge sharing (knowledge acquisition and knowledge contribution), and innovation performance. | Interpersonal trust was measured following McAllister (1995). Innovation performance scale was  measured based on a published study involving innovation effectiveness and innovation efficiency. | Interpersonal | 209 members of 4-8-person teams | Interpersonal trust has a positive effect on both innovation performance and its dimensions, and knowledge sharing partially mediates the role of interpersonal trust in influencing innovation performance. |
| 17 | Wang & Cai, 2016 | Parental leadership (divided into virtuous, benevolent and authoritarian leadership), innovative performance of teachers’ teaching, trust, and autonomous motivation. | A trust scale from Mascall et al. (2009) and  teacher innovation performance scale cited from a published study were applied. | Interpersonal | 1123 teachers from 40 schools in Hebei and Shanxi | Virtuous and benevolent leadership promote innovative teaching performance and authoritarian leadership inhibits innovative teaching performance; paternalistic leadership behaviors positively influence autonomous motivation and ultimately affect innovative teacher teaching performance by affecting feelings of trust. |
| 18 | Bidault & Castello, 2009 | Trust, creativity (percentage difference between one’s individual creativity and the creativity of the pair), and personality (as control variables) | Trust games were completed by two persons divided into a group; creativity (percentage difference between one individual’s creativity and the creativity of the pair) was measured through a cognitive task. | Interpersonal | 108 college students from five business schools | The amount of mutual trust between partners that maximizes joint creativity and innovation appears to have an inverted (positive) U-shaped association. |
| 19 | Klimoski & Karol 1976 | Trust and creative problem solving. | Interpersonal trust was induced via manipulated feedback from three other group members, whereas creativity was measured through a standard brainstorming task, a self-report task, and synaptics. | Interpersonal | 29 four-person groups of undergraduate students | Results show that the high-trust and control groups outperformed the low-trust group in all three tasks. |
| 20 | Chua, Morris, & Mor, 2012, the pilot study of study 3 | Cultural metacognition, intercultural trust, and creative collaboration. | Intercultural trust was measured with the following question, “Did the negotiation make you trust your counterpart?”; creative collaboration was measured with the following question, “Based on your interaction with your counterpart in this negotiation exercise, to what extent is he or she a good partner to work with on future projects that require considerable innovation and creativity?” | Interpersonal | 76 MBA students | Cultural metacognition has a positive relationship to trust, which in turn has a positive relationship with creative collaboration. |
| 21 | Chua, Morris, & Mor, 2012, the main study of study 3 | Cultural metacognition and creative collaboration. | Cognition- and affect-based trust were measured using three items each (adapted from McAllister, 1995); creative collaboration was measured by three items on whether the other person was a good partner for creative work evaluated on a 7-point scale. The three items were set as follows: (a) “How interested are you in working on another creativity task with your partner if given a chance to do so in the future?”; (b) “Overall, how would you rate your partner’s creativity?”; and (c) “To what extent is he or she a good partner to work with on projects that require considerable innovation and creativity?” | Interpersonal | 236 students (118 dyads) | Affect- and cognition-based trust are positively correlated with perceptions of one’s partner’s creative collaboration in sharing ideas within the dyad. Importantly, affect-based trust not only partially mediates the effect of cultural metacognition on collaborative creative performance and perceptions of one’s partner as effective at creative work but also fully mediates the effect of metacognition on the sharing of ideas. |
| 22 | Levin & Cross, 2004 | Receipt of useful knowledge, tie strength, tacit knowledge, benevolence-based trust, and competence-based trust. | The receipt of useful knowledge was measured by four items as the creativity indicator. | Interpersonal | 138 employees from three companies | The positive impact of strong ties on effective knowledge transfer is mediated by competence- and benevolence-based trust; competence-based trust is especially important for the transfer of tacit knowledge. |
| 23 | Carmeli & Spreitzer, 2009 | Innovative work behaviors, thriving, connectivity, and trust in one’s employer. | Innovative work behaviors were measured using the 6-item scale developed by Scott and Bruce (1994); trust was measured by the four-item scale developed by Robinson (1996). | Interpersonal | 172 employees were measured at two points in time with a lag of three weeks between Time 1 and Time 2 | Innovative work behavior has a positive relationship with trust for employers, resulting in a sequentially mediated model in which connectivity mediates the relationship between trust and prosperity and prosperity mediates the relationship between connectivity and innovative work behavior. |
| 24 | Zhang, Zhou, & Wang, 2012 | Internal and external social networks of the company, team trust, and company innovation capacity. | The trust of entrepreneurial team members was measured as the extent to which team members are happy to share knowledge and information without fearing opportunistic behavior from other members measured on a 6-item scale.  Entrepreneurial innovativeness was measured with the Cengiz Yilmaz scale. | Group | 112 enterprises | The higher the level of trust among the members of an entrepreneurial team is, the greater the positive impact of the internal social network on the innovation capacity of the firm will be. |
| 25 | Shi, Wang, & Deng, 2015 | Executive team heterogeneity, executive team trust, and innovation strategy. | Team trust was measured following MCAllister (1995). A self-developed innovation strategy selection questionnaire with inherited and disruptive innovation dimensions (3 items each) was applied. | Group | 203 companies | Relationship-oriented trust has a positive effect on inherited innovation strategies. Job-oriented trust has a positive relationship with disruptive innovation and is positively moderated by executive team heterogeneity. |
| 26 | Shan, Yu, & Lu, 2019 | Employee innovative behavior, perceived entrepreneurial passion, and employee perceived trust. | Employee innovative behavior was measured following Scott & Bruce (1994).  Employee perceived trust was measured following Lau, Lam, & Wen (2014). | Group | 122 new companies founded less than 10 years ago | There is a significant correlation between employee perceived trust and employee innovation behavior; both employee perceived passion for exploration and passion for developing new ventures positively influence employee innovation behavior through perceived trust. |
| 27 | Chen & Pan, 2015 | Partnership, trust, explicit knowledge sharing, tacit knowledge sharing, and innovation performance. | Two scales individually measuring trust and creativity were adopted from previously published studies. | Group | 256 companies | Trust has a positive effect on both explicit and tacit knowledge sharing. |
| 28 | Yang, Yang, & Ma, 2013 | Collectivist culture, individualist culture, trust level, trust approach (including cognitive and emotional trust), and technological innovation approach. | Trust level was measured following Simons et al. (2000); trust was measured following McAllister (1995), including cognitive and affective trust.  Technological innovation modes were measured following a published study measuring 9 items for mutational and incremental innovation. | Group | 173 manufacturing and high-tech enterprises | A collectivist culture promotes mutational innovation, and the combination of collectivist and affective trust promotes mutational innovation; an individualist culture promotes incremental innovation, and the combination of individualist and cognitive trust promotes incremental innovation; trust levels have a positive moderating effect on the relationship between collectivist and mutational innovation. |
| 29 | Chen & Pan, 2015 | Social relationships, trust, knowledge sharing among supply chain companies (including explicit and tacit knowledge sharing), and innovation performance. | Trust was measured following Seppanen (2007).  Innovation performance was measured following Jantunen (2005). | Group | 256 upstream and downstream supply chain companies | Social relationships positively affect tacit knowledge sharing.  Trust has a positive impact on explicit and implicit knowledge sharing, which both have a positive impact on innovation performance. |
| 30 | Yang & Chen, 2019 | Team trust, team innovation performance, information refinement, and team reflection. | Team trust was measured with trust scales from McAllister (1996) and Dirks (2001). Team innovation performance was measured following West's (1998) one-dimensional scale. | Group | 324 persons from 46 high-tech enterprise teams | Team trust positively influences team innovation performance; information refinement plays a mediating role in this relationship, and team reflection plays a positive moderating role in this relationship. |
| 31 | Brattström, Löfsten, & Richtnér, 2012 | Trust (goodwill and competence), creativity, and systematic processes and structures of product development. | All measures were measured on Likert-type scales selected from previous studies ranging from 1 (strongly disagree) to 5 (strongly agree). | Group | 192 firms  99 firms | Two constructs of trust are both positively correlated with creativity, showing that trust increases creativity with goodwill trust serving as a mediating variable in the association between combined systematic processes and structures and creativity. |
| 32 | Akhtar, Khan, Hassan, Irfan, & Atlas, 2019 | Transformational leadership, team communication, team trust, team creativity, and task performance. | The five items of team trust were adopted from Barczak et al. (2010), Cook and Wall (1980), and McAllister (1995). Items of team creativity were adopted from multiple published studies. | Group | 273 college students | Team trust improves team creativity. |
| 33 | Liu, 2020 | Swift trust, interactive behaviors (perceptions of teamwork competence), and team creativity. | Perceptions of both cognition- and affect-based swift trust were measured with a swift trust scale developed for multinational teams of MBA students developed by Kanawattanachai and Yoo (2002); team creativity was measured with the 10-item team creativity scale developed by Yang et al. (2010). | Group | 210 college students majoring in nursing or design | Among nursing students who belong to an interdisciplinary student team, there is a positive correlation between cognitive-based team swift trust and team creativity, with helping behaviors and spontaneous communication indirectly mediating the relationship between cognitive-based team swift trust and team creativity. |
| 34 | Boies, Fiset, & Gill, 2015 | Motivation, team communication, team trust, and team creativity. | Team trust was assessed using five items from Cook and Wall's (1980) scale; creativity tasks were developed based on Woolley (2009) and required teams to construct highly intricate structures from building blocks. | Group | 44 teams with 2-4 students totaling 137 persons | A positive correlation between novelty but not usefulness and trust was found with trust serving as the mediating variable of the relationship between communication and creativity. |
